# Causal relationships between 4907 circulating proteins and heart failure and atrial fibrillation: A bidirectional Mendelian randomization study

**DOI:** 10.1097/MD.0000000000043198

**Published:** 2025-07-04

**Authors:** Zhenyu Yang, Xiaohan Xiu, Fengzhao Liu, Jixin Li, Zheng Tang, Weibo Zhong, Shoulei Xu, Shuo Ma, Yating Wang, Ruixue Zuo, Dandan Guo

**Affiliations:** a Heilongjiang University of Chinese Medicine, Harbin, China; b Xiyuan Hospital, China Academy of Chinese Medical Sciences, Beijing, China; c The First Affiliated Hospital of Chongqing Medical University, Chongqing, China; d Shanghai University of Traditional Chinese Medicine, Shanghai, China; e The Second Affiliated Hospital of Heilongjiang University of Chinese Medicine, Harbin, China.

**Keywords:** atrial fibrillation, circulating proteins, enrichment analysis, heart failure, mediation, Mendelian randomization

## Abstract

The 5-year survival rate of patients with heart failure (HF) is comparable to that of malignant tumors, with a persistently high risk of mortality. Atrial fibrillation (AF) is a common cardiac arrhythmia, with its incidence rising annually due to the aging population. Therefore finding relevant therapeutic targets for both diseases is crucial. Firstly, instrumental variables were obtained from publicly available genome-wide association study databases. Secondly, 5 robust Mendelian randomization (MR) methods were employed to assess causal effects between exposure and outcome. Thirdly, we also performed multiple sensitivity analyses and corrected all inverse variance weighted results for false discovery rate (FDR). Fourthly, reverse MR analysis and Mediation MR analysis can improve the completeness of the study. Finally, the protective proteins were analyzed for pathway enrichment and potential drug targets were explored via the DrugBank website. This study identified a total of 23 circulating proteins associated with the diseases, among which defensin beta 135 (DEFB135, *P*_IVW_ < .001, *P*_FDR_ = .017) emerged as a shared protective protein against both diseases. Conversely, reverse MR analysis revealed no statistically significant impact of the diseases on these circulating proteins. Mediation MR analysis identified AF as the mediator between DEFB135 and HF. Enrichment analysis elucidated a series of pathways related to HF and AF. Finally, a total of 8 proteins were retrieved from the database. Our MR analysis identified 23 circulating proteins associated with the diseases, providing valuable references for clinical treatment.

## 1. Introduction

Currently, approximately 40% of global mortality is attributed to cardiovascular diseases, making them the leading cause of death worldwide.^[1]^ Heart failure (HF) is characterized by impaired cardiac structure and function, resulting in inadequate cardiac output to meet the metabolic demands of the body’s tissues, representing the terminal stage of most cardiovascular diseases.^[2]^ With the prevalence of unhealthy lifestyles and the exacerbation of population aging, the incidence of HF is on the rise.^[3]^ Atrial fibrillation (AF) is a common rapid cardiac arrhythmia associated with a significantly increased risk of diseases such as stroke, HF, and myocardial infarction, imposing a substantial economic burden on society.^[4]^ The annual incidence rate of HF in AF patients is 33%, leading to a frequent coexistence of these 2 conditions, ultimately forming a vicious cycle.^[5,6]^

Circulating proteins, as key regulatory factors in molecular pathways, play pivotal roles in various biological processes (BPs) and are often regarded as targets for drug therapy.^[7]^ Currently, research on circulating proteins in the cardiovascular field is expanding. Surfactant protein type B (SPB) is essential for gas exchange in the alveoli. A study involving 80 chronic HF patients and 20 healthy controls demonstrated that SPB is associated with gas exchange and ventilation efficiency, with its concentration positively correlated with the severity of HF, confirming SPB as a biomarker for lung alveolar-capillary barrier damage in chronic HF patients.^[8]^ A serum proteomic analysis revealed that *β*-2-microglobulin, dystroglycan, and other 5 highly sensitive and specific proteins could serve as potential biomarkers for HF patients with phlegm-blood stasis syndrome.^[9]^ Another study found an increased risk of AF associated with decreased levels of insulin-like growth factor 1 and elevated levels of insulin-like growth factor-binding protein 1.^[10]^ Although research on circulating proteins is expanding, most studies are limited to a few protein species or small sample sizes. It is worth noting that these studies are predominantly observational and suffer from design flaws. The inability to exclude confounding factors and reverse causality interference renders the study results insufficient.

Mendelian randomization (MR) addresses these issues. MR is a popular and robust analytical method that simulates randomized controlled trials by using randomly allocated single nucleotide polymorphisms (SNPs) as instrumental variables (IVs) to infer causal relationships between exposure and related outcomes. Compared to traditional randomized controlled trials (RCTs), MR is efficient and cost-effective, requiring minimal financial and time investment and avoiding ethical concerns, making it more suitable for large-scale screening of causal relationships. With the continuous development of genome-wide association studies (GWAS) on the human circulating proteome, an increasing number of researchers have used MR studies to confirm the causal relationships between circulating proteins and diseases such as stroke,^[11]^ primary biliary cholangitis,^[12]^ type 2 diabetes,^[13]^ and acute pancreatitis.^[14]^ Inspired by the aforementioned studies, this study employs the largest-scale circulating proteome to date to evaluate the causal relationships between HF and AF and explore potential targets for their treatment.

## 2. Methods

### 2.1. Basic approach of the study

**Figure 1.**
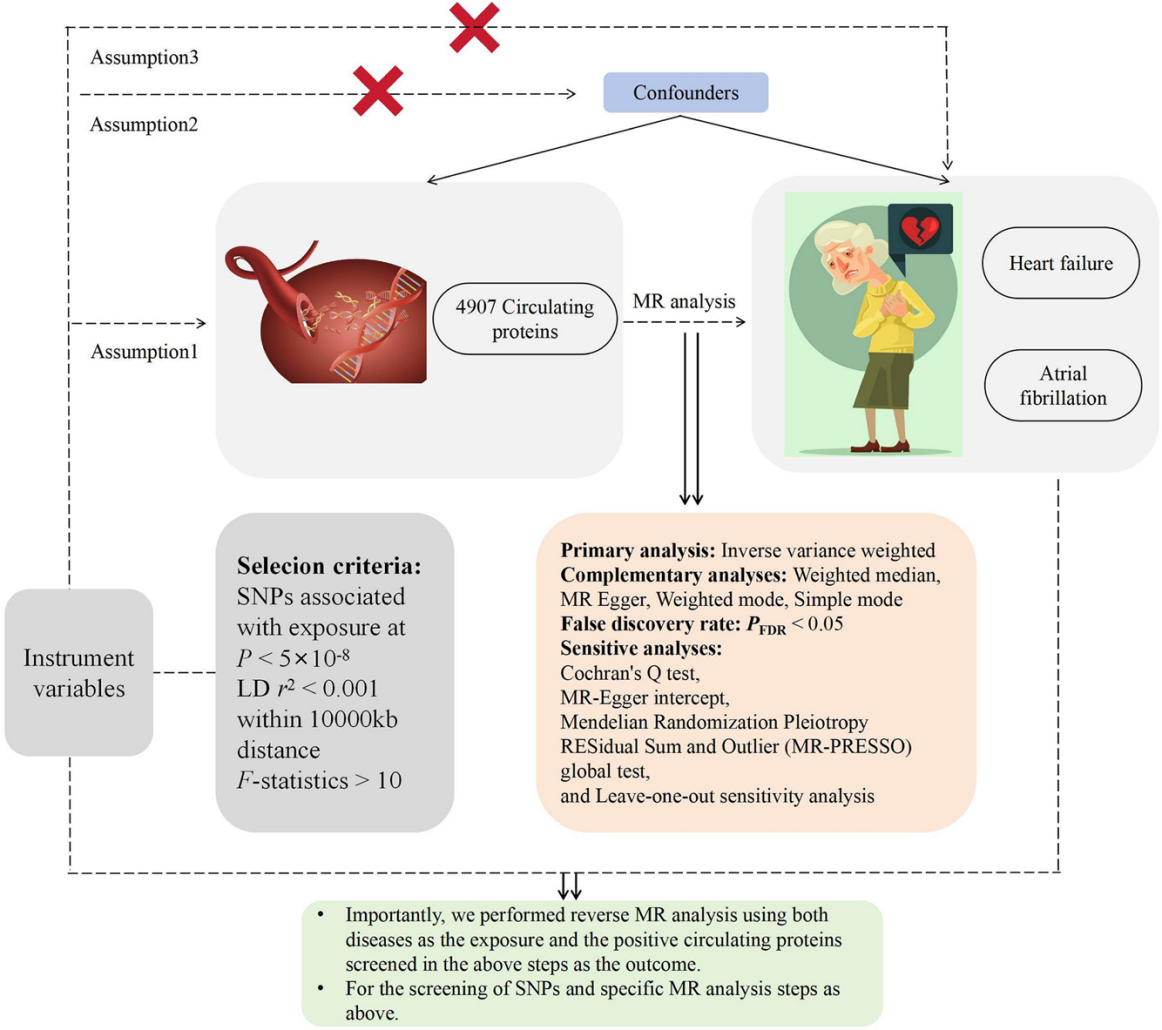
The basic idea map of this study.

The fundamental approach of this study is illustrated in Figure 1. In this study, 4907 circulating proteins were considered as exposures, while HF and AF were treated as outcomes, and their causal relationships were assessed through MR analysis. In the reverse MR analysis, the selected positive proteins were regarded as outcomes, with HF and AF as exposures, to evaluate reverse causality. The basic principles to be followed in MR studies are as follows: IVs should be closely associated with the exposure factor; IVs should be independent of any confounding factors related to exposure and outcome; IVs should affect the outcome only through the exposure factor and not directly impact the outcome.

### 2.2. Data sources

The proteomic GWAS data used in this study were extracted from a large-scale protein quantitative trait loci (pQTL) study involving 35,559 Icelandic individuals.^[15]^ This study identified a total of 4907 circulating proteins and reported summary statistics for over 270 million genetic variants. Genomics and proteomics are interconnected, where pQTLs represent specific manifestations of genetic loci at the protein level, thus facilitating the assessment of causal relationships between circulating proteins and diseases and the screening of therapeutic targets. Summary data for HF (GWAS ID: ebi-a-GCST009541, https://gwas.mrcieu.ac.uk/datasets/ebi-a-GCST009541/) and AF (GWAS ID: ebi-a-GCST006061, https://gwas.mrcieu.ac.uk/datasets/ebi-a-GCST006061/) were obtained from the HERMES Consortium. The HF GWAS data included 977,323 individuals of European descent (ncase = 47,309, ncontrol = 930,014) and 7773,021 SNPs. The AF study comprised 537,409 European individuals, including ncase = 55,114, ncontrol = 482,295, with a total of 12,095,506 SNPs. The case group for HF included participants clinically diagnosed with HF of any etiology, while the control group comprised participants without HF; the case group for AF was patients with clinical symptoms, physical examination and electrocardiograms suggestive of AF, and the control group was participants without AF. All data sources were approved by the appropriate ethical review boards and no additional ethical approvals were required as only summary-level statistics were used in this study. For further details on GWAS, please refer to the original publications.^[16,17]^

### 2.3. Selection of instrumental variables

To meet the assumptions of MR and select qualified IVs, a series of strict selection criteria were established. Firstly, the genome-wide significance level was set at *P* < 5 × 10^−8^ to filter out a large number of SNPs unrelated to the exposure factor. Secondly, to eliminate interference from SNPs in linkage disequilibrium, SNPs with *r*^2^ < 0.001 within a range of kilobase pairs (kb) = 10,000 were excluded. Then, to mitigate bias from weak IVs, *F* statistics were calculated, and only IVs with higher *F* statistics (>10) were included in subsequent analyses.^[18]^ At the same time, SNPs with echo structures should be excluded when coordinating exposures and endings using the harmonise_data function. Finally, the Pheno Scanner V_2.0_ database was utilized to exclude SNPs related to potential confounding factors such as smoking, alcohol consumption, hypertension, obesity, and physical activity. It is noteworthy that before conducting MR analysis, we ensure that each SNP has *P*_outcome_ > *P*_exposure_, ensuring that all IVs affect the outcome only through exposure, thus meeting the exclusivity assumption of MR.

### 2.4. Statistical analysis

#### 2.4.1. Mendelian randomization analysis

We conducted MR analysis using 5 methods from the TwoSampleMR package in R 4.3.2 to assess the causal relationships between circulating proteins and HF and AF. The inverse variance weighted (IVW) method, being able to consider the influence of various confounding factors and estimate the combined effects of multiple genetic variants on the disease, is the primary analysis method.^[19]^ MR-Egger is a method that can be used for causal inference and sensitivity analysis. When there are many invalid IVs or potential pleiotropy in the study results, the analysis results of MR-Egger are more reliable.^[20]^ Weighted median is also a common method for MR analysis, which can provide credible effect estimates even if up to half of the IVs are invalid.^[21]^ It serves as a complement to IVW and MR-Egger. Simple mode and weighted mode, compared to the first 3 methods, have lower statistical power because they are 2 methods that further relax the assumptions of MR studies.^[22]^ When different analysis methods yield different conclusions, IVW method should be given priority.

#### 2.4.2. Sensitivity analysis

For sensitivity analysis, we conducted the following analyses. The MR Pleiotropy RESidual Sum and Outlier (MR-PRESSO) global test was used to identify and remove horizontal pleiotropic outliers, thereby improving the accuracy of the study results.^[23]^ However, at least half of the IVs are valid hypotheses for MR-PRESSO to give accurate estimates. When *P* < .05 for the MR-PRESSO global test, there are horizontal multidimensional outliers that need to be eliminated; conversely, there are no horizontal multidimensional outliers. Then, we performed heterogeneity analysis of the study results using Cochran *Q* analysis, and verified whether genetic variants have directional pleiotropy through the MR-Egger intercept test. It is important to note that the presence of pleiotropy in study results is absolutely not allowed and must be eliminated; however, a small amount of genetic heterogeneity does not affect the reliability of the results, which is merely due to the large differences between different SNPs. Leave-One-Out is also a method of sensitivity analysis, which tests the stability of the results by removing each SNP from the exposure, while funnel plots are used to check for bias in the results. Finally, to reduce false positive results caused by multiple comparisons, we corrected all results using the false discovery rate (FDR) method, ensuring *P*_IVW_ < .05 and *P*_FDR_ < .05.

#### 2.4.3. Visualization

To enhance the credibility of the study, we included only circulating proteins with consistent direction of odds ratio (OR) values across the 5 MR analysis methods. For disease-related proteins, we plotted volcano maps (green dots representing protective factors, red dots representing risk factors). To visually display the results of MR analysis more intuitively, we generated scatter plots, where the 5 sloping lines represent the 5 different analysis methods, and the slope of the line demonstrates whether the exposure is a protective or risk factor for the disease. Finally, by plotting forest plots, we can observe the extent to which each SNP affects the results.

#### 2.4.4. Reverse MR analysis

The focus of this study was on the impact of circulating proteins on disease, and the reverse MR analysis was only a complementary study to the main results. Using disease-related proteins as outcomes and HF and AF as exposures, we conducted MR analysis again to observe the impact of diseases on circulating proteins. The selection of IVs and specific analysis methods were the same as above.

#### 2.4.5. Mediation MR analysis

After MR analysis, exploring whether 1 disease plays a mediating role between circulating proteins and another disease would be an interesting research direction. For this study, the prerequisite for mediation MR analysis is that circulating proteins have statistically significant effects on both AF and HF, but diseases have no statistically significant effects on circulating proteins. At the same time, the effect of 1 disease on another disease is statistically significant.

#### 2.4.6. Enrichment analysis

To further investigate the complex relationships and biological functions of the important proteins we discovered, we conducted enrichment analysis of protective proteins using the clusterProfiler package in R 4.3.2 and the gene ontology (GO) and Kyoto encyclopedia of genes and genomes (KEGG) databases. The GO database divides genes into 3 parts according to BP, cellular component (CC), and molecular function (MF). Additionally, we visualized the results of enrichment analysis by plotting circle charts, bar charts, and bubble charts. For circle charts, from the outside to the inside, the different pathways, the number of genes enriched in each pathway, the number of associated proteins enriched in each pathway, and the proportion of genes. In bar charts, the horizontal coordinate represents the number of proteins, the vertical coordinate labels the name of each pathway, and the color of the bar represents significance. Unlike bar charts, the horizontal coordinates in bubble charts represent the proportion of genes, and the size of the bubbles represents the number of proteins.

#### 2.4.7. Drug target analysis

The DrugBank website (https://go.drugbank.com/) provides detailed information on drug names and development processes. Given the potential of circulating proteins to become emerging drug targets, we searched for disease-related proteins identified in our study using the DrugBank website. These proteins were categorized into 3 types: approved (one or more drugs targeting the protein have been approved); under investigation (targeting drugs are currently in clinical research stage); potential drug targets (proteins not found in the database are considered potential drug targets).

## 3. Results

### 3.1. Disease-related proteins

**Figure 2.**
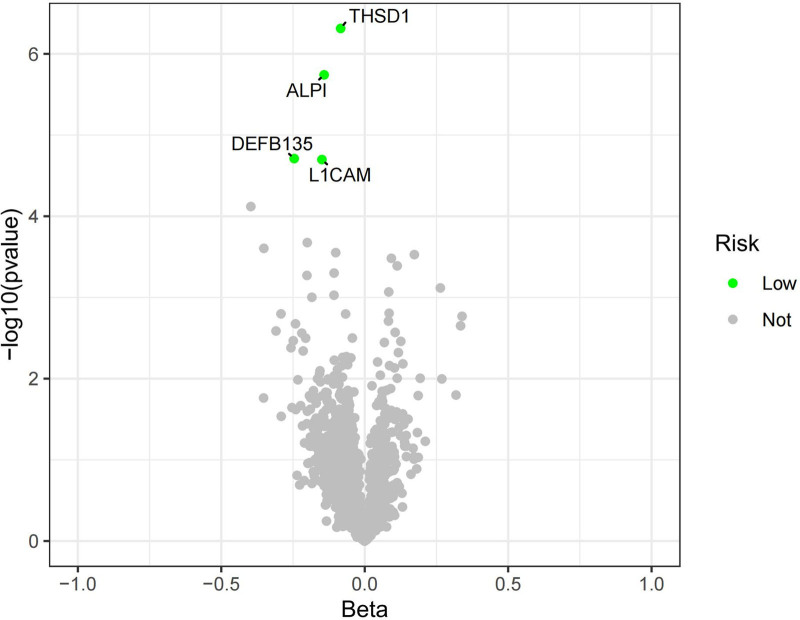
Volcano map of HF. HF = heart failure.

**Figure 3.**
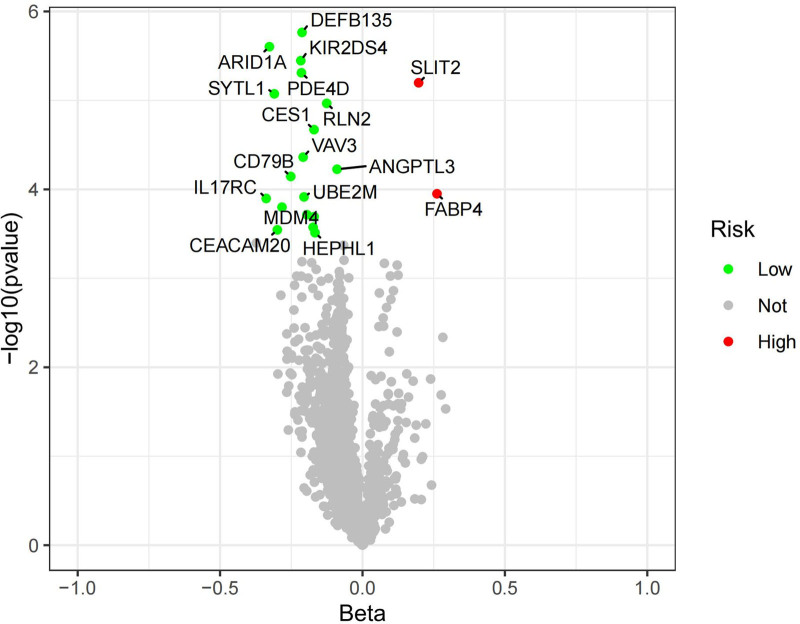
Volcano map of AF. AF = atrial fibrillation.

After FDR correction, 21 circulating proteins were considered associated with AF (*P*_IVW_ < .05, *P*_FDR_ < .05), and 4 circulating proteins were considered associated with HF (*P*_IVW_ < .05, *P*_FDR_ < .05), as shown in Table 1. Following a search in the DrugBank database, it was found that 15 proteins were not retrieved, indicating the novelty of our study, and they are considered potential drug targets. The volcano plots for HF and AF outcomes are presented in Figures 2 and 3, respectively, indicating SLIT2 and FABP4 as risk factors for AF. In this study, the range of IVs included when circulating proteins were considered exposures was 3 to 12, with all *F* statistics >10, and no horizontal pleiotropic outliers were detected by the MR-PRESSO global test, as detailed in Tables S1 to S24, Supplemental Digital Content, https://links.lww.com/MD/P365.

**Table 1:** Disease-associated circulating proteins.

Exposure	Targets	*P*_IVW_	*P*_FDR_	Outcome	Id.outcome
THSD1	Tier 3 targets	<.001	.002	Heart failure	ebi-a-GCST009541
ALPI	Tier 2 targets	<.001	.003	Heart failure	ebi-a-GCST009541
DEFB135	Tier 3 targets	<.001	.017	Heart failure	ebi-a-GCST009541
L1CAM	Tier 3 targets	<.001	.017	Heart failure	ebi-a-GCST009541
DEFB135	Tier 3 targets	<.001	.004	Atrial fibrillation	ebi-a-GCST006061
ARID1A	Tier 3 targets	<.001	.004	Atrial fibrillation	ebi-a-GCST006061
KIR2DS4	Tier 3 targets	<.001	.004	Atrial fibrillation	ebi-a-GCST006061
PDE4D	Tier 1 targets	<.001	.004	Atrial fibrillation	ebi-a-GCST006061
SLIT2	Tier 3 targets	<.001	.004	Atrial fibrillation	ebi-a-GCST006061
SYTL1	Tier 3 targets	<.001	.005	Atrial fibrillation	ebi-a-GCST006061
RLN2	Tier 3 targets	<.001	.005	Atrial fibrillation	ebi-a-GCST006061
CES1	Tier 1 targets	<.001	.008	Atrial fibrillation	ebi-a-GCST006061
VAV3	Tier 3 targets	<.001	.015	Atrial fibrillation	ebi-a-GCST006061
ANGPTL3	Tier 1 targets	<.001	.018	Atrial fibrillation	ebi-a-GCST006061
CD79B	Tier 3 targets	<.001	.020	Atrial fibrillation	ebi-a-GCST006061
FABP4	Tier 1 targets	<.001	.027	Atrial fibrillation	ebi-a-GCST006061
UBE2M	Tier 3 targets	<.001	.027	Atrial fibrillation	ebi-a-GCST006061
IL17RC	Tier 3 targets	<.001	.027	Atrial fibrillation	ebi-a-GCST006061
CASK	Tier 1 targets	<.001	.032	Atrial fibrillation	ebi-a-GCST006061
HERC4	Tier 3 targets	<.001	.036	Atrial fibrillation	ebi-a-GCST006061
RND1	Tier 2 targets	<.001	.036	Atrial fibrillation	ebi-a-GCST006061
MDM4	Tier 3 targets	<.001	.045	Atrial fibrillation	ebi-a-GCST006061
CEACAM20	Tier 3 targets	<.001	.046	Atrial fibrillation	ebi-a-GCST006061
HEPHL1	Tier 1 targets	<.001	.047	Atrial fibrillation	ebi-a-GCST006061

ALPI = intestinal-type alkaline phosphatase, ANGPTL3 = angiopoietin like protein 3, ARID1A = AT-rich interaction domain 1A, CASK = calcium/calmodulin dependent serine protein kinase, CD79B = cluster of differentiation 79B, CEACAM20 = carcinoembryonic antigen related cell adhesion molecule 20, CES1 = carboxylesterase 1, DEFB135 = defensin Beta 135, FABP4 = fatty acid binding protein 4, FDR = false discovery rate, HEPHL1 = hephaestin-like protein 1, HERC4 = E3 ubiquitin-protein ligase HERC4, IL17RC = interleukin 17 receptor C, IVW = inverse variance weighted, KIR2DS4 = killer cell immunoglobulin-like receptor 2DS4, L1CAM = L1 cell adhesion molecule, MDM4 = murine double minute 4, PDE4D = phosphodiesterase 4D, RLN2 = relaxin 2, RND1 = rho family GTPase 1, SLIT2 = slit guided ligand 2, SYTL1 = synaptotagmin-like protein 1, THSD1 = thrombospondin type-1 domain-containing protein 1, UBE2M = ubiquitin conjugating enzyme E2 M, VAV3 = guanine nucleotide exchange factor VAV3.

### 3.2. MR analysis results

**Figure 4.**
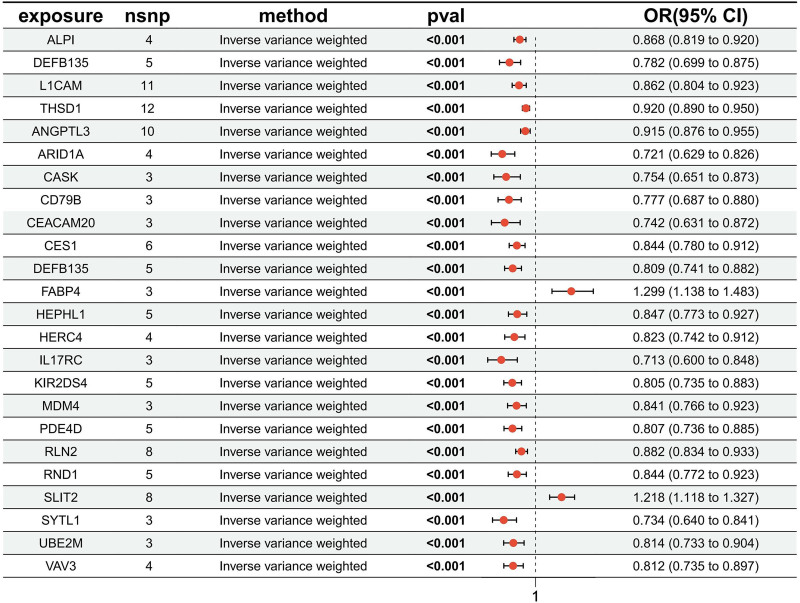
Analytical results of the MR study. MR = Mendelian randomization.

Five robust MR analysis methods were employed to assess the impact of circulating proteins on diseases, with consistent OR values across all 5 methods, and statistically significant IVW method (*P*_IVW_ < .05). Figure 4 illustrates the *P*-values, OR values, and 95% confidence intervals for the IVW method. Notably, the first 4 rows in the figure represent the results of MR analysis with HF as the outcome. As shown in Table S25, Supplemental Digital Content, https://links.lww.com/MD/P366, the MR-Egger intercept test indicated no directional pleiotropy in genetic variants, and Cochran *Q* analysis revealed no significant genetic heterogeneity among the included SNPs. Sensitivity analysis using leave-one-out found no SNPs exerting substantial influence on the final results, while funnel plots indicated a uniform distribution of SNPs for all MR analyses, suggesting no significant bias in the study results. Visualization results of MR analysis are provided in Figures S1 to S48, Supplemental Digital Content, https://links.lww.com/MD/P367.

### 3.3. Reverse MR analysis and mediation MR analysis results

When considering AF or HF as exposures and disease-related proteins as outcomes, we found that neither AF nor HF had statistically significant effects on these disease-related proteins. This, on the contrary, enhances the significance of the positive MR study, showing that neither AF nor HF will have an interfering effect on these circulating proteins, and at the same time confirms the possibility that these circulating proteins have the potential to be a target for medication from another perspective.

**Figure 5.**
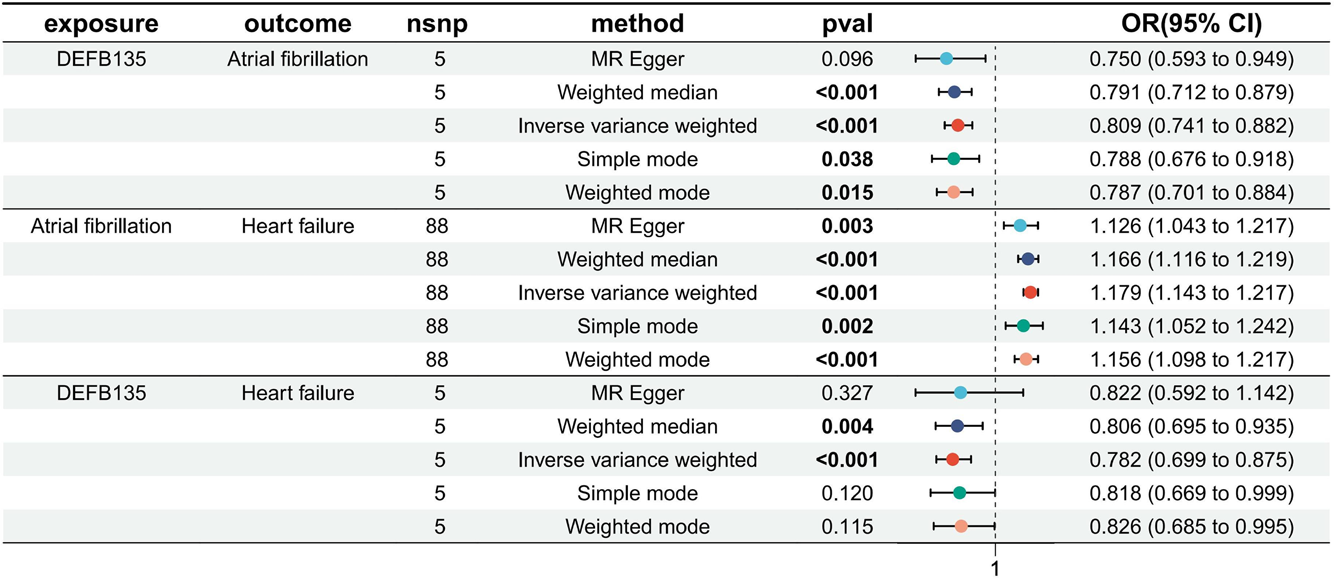
Mediation MR analysis. MR = Mendelian randomization.

Of all the disease-associated proteins, only DEFB135 had a statistically significant effect on both AF and HF. We used DEFB135 as the exposure, AF and HF as the mediating variables, respectively, and another disease as the outcome, and performed MR analyses according to the strict statistical methods specified above. Ultimately, the effect on HF was found to be statistically significant only when AF was used as a mediating variable. DEFB135 reduced the risk of HF through AF mediation, with a mediated effect of −0.035 (−0.058 to −0.012), a mediated proportion of 14.200% (4.730%, 23.800%), and *P* = .003, as depicted in Figure 5.

### 3.4. Enrichment analysis results

**Figure 6.**
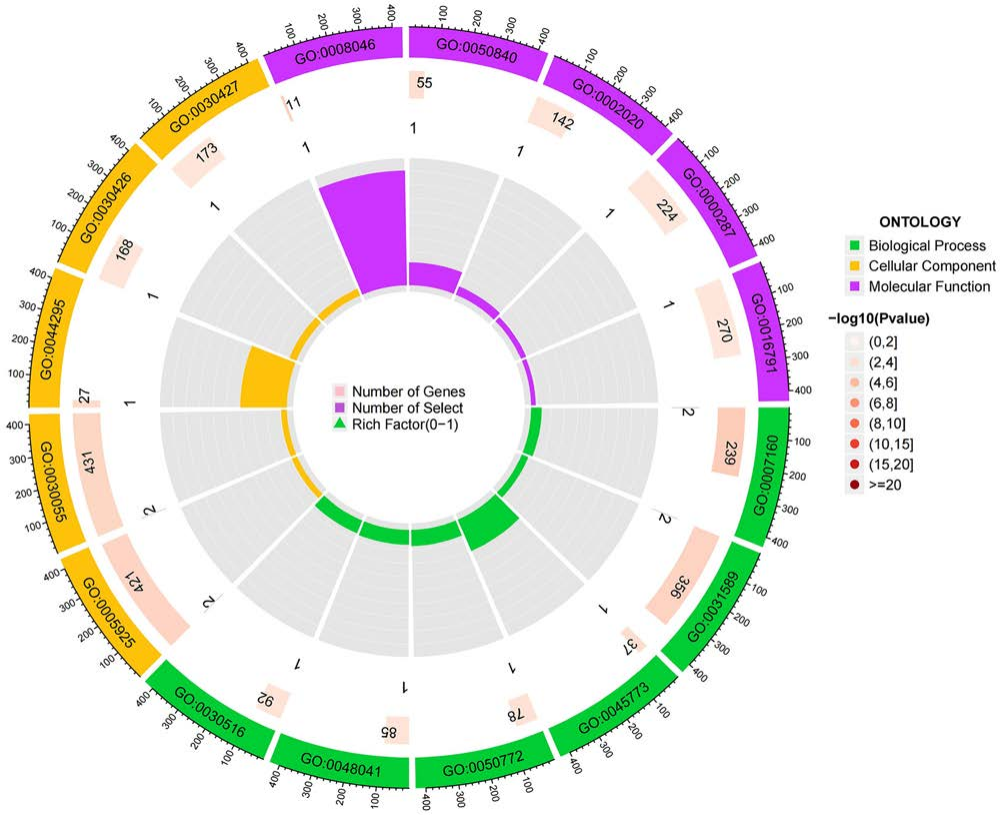
Circle chart for GO enrichment analysis HF. GO = gene ontology, HF = heart failure.

**Figure 7.**
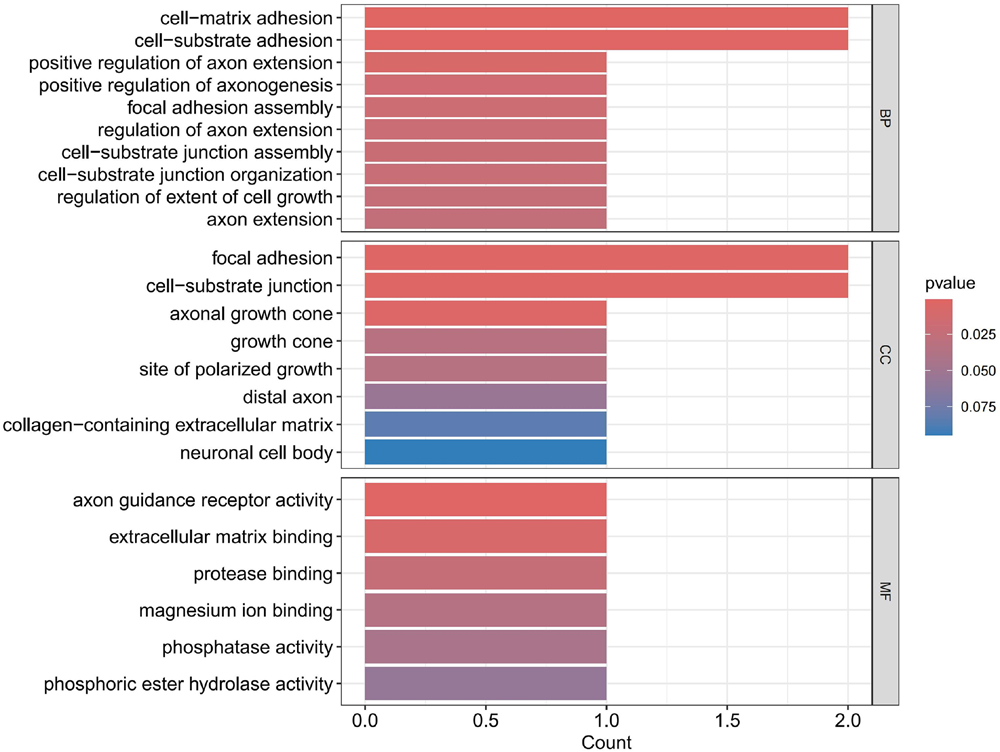
Bar chart for GO enrichment analysis HF. GO = gene ontology, HF = heart failure.

**Figure 8.**
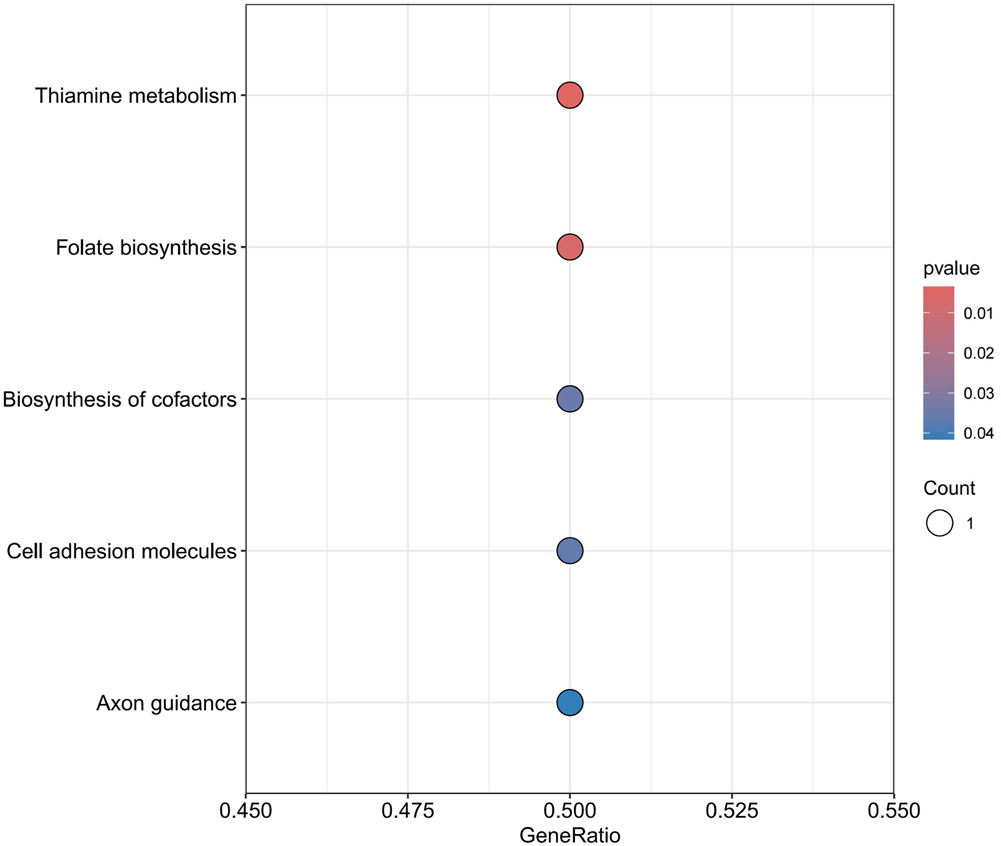
Bubble chart for KEGG enrichment analysis HF. HF = heart failure.

For the protective circulating proteins, we conducted pathway enrichment analysis using the GO and KEGG databases. In terms of BP, proteins associated with HF were found to be enriched in cell-matrix adhesion, cell-substrate adhesion, and positive regulation of axon extension. Regarding CC, these proteins were mainly associated with focal adhesion, cell-substrate junction, and axonal growth cone pathways. As for MF, the enrichment was observed in axon guidance receptor activity, extracellular matrix binding, and protease binding pathways. KEGG enrichment analysis revealed enrichment of proteins associated with HF in Thiamine metabolism, Folate biosynthesis, Biosynthesis of cofactors, Cell adhesion molecules, and Axon guidance pathways, as detailed in Figures 6 to 8.

**Figure 9.**
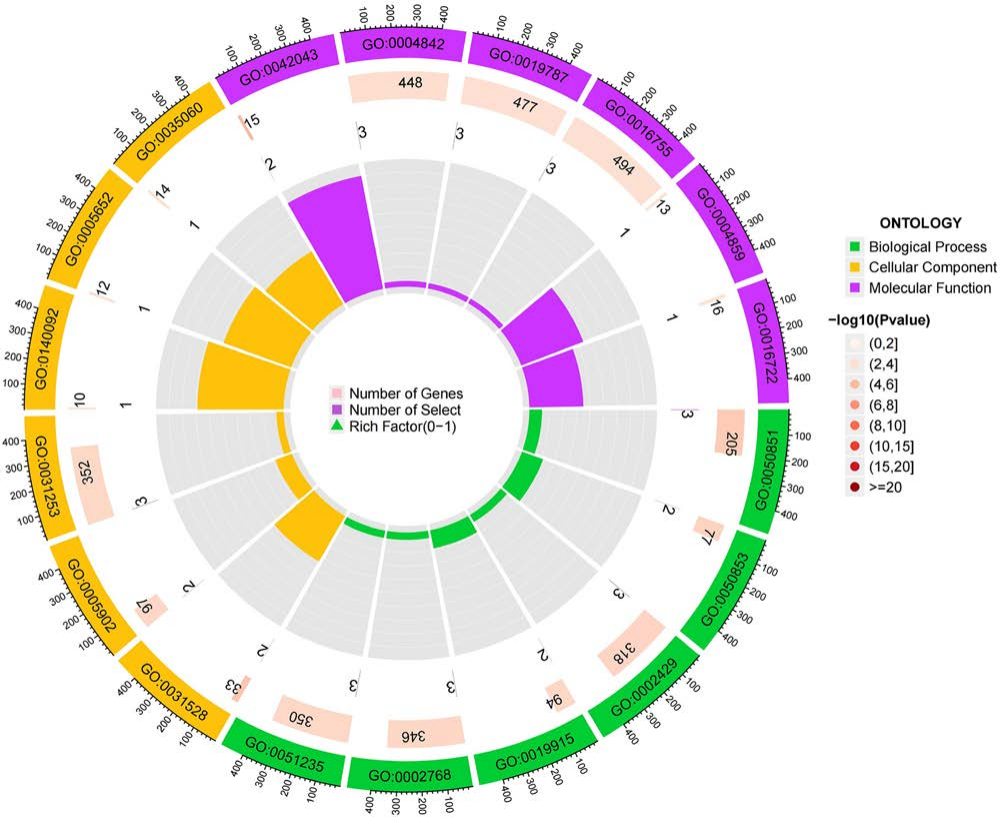
Circle chart for GO enrichment analysis AF. AF = atrial fibrillation, GO = gene ontology.

**Figure 10.**
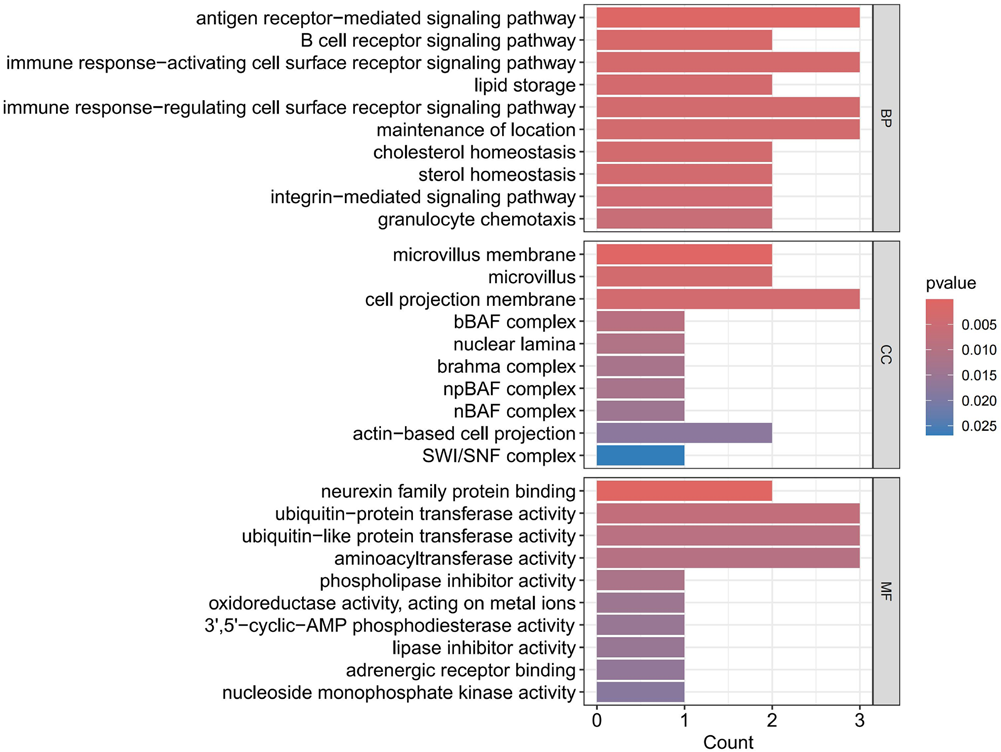
Bar chart for GO enrichment analysis AF. AF = atrial fibrillation, GO = gene ontology.

**Figure 11.**
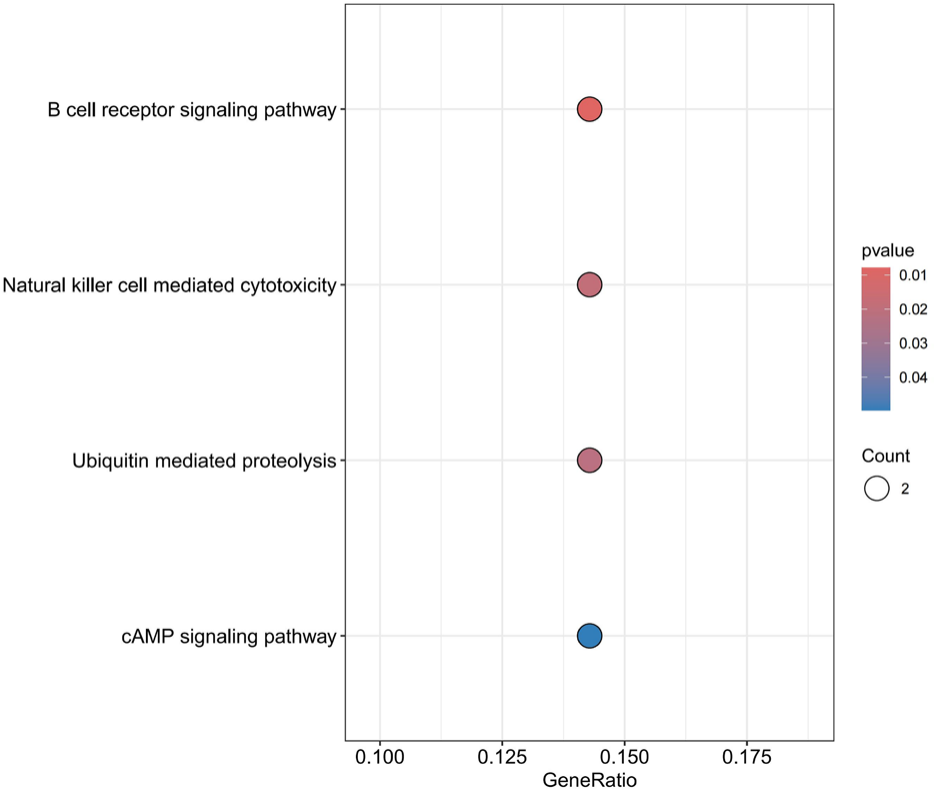
Bubble chart for KEGG enrichment analysis AF. AF = atrial fibrillation, KEGG = Kyoto encyclopedia of genes and genomes.

As depicted in Figures 9 to 11, when AF was considered as the outcome, in terms of BP, protective proteins were found to be enriched in antigen receptor-mediated signaling pathway, B cell receptor signaling pathway, and immune response-activating cell surface receptor signaling pathway. Regarding CC, these proteins were mainly associated with microvillus membrane, cell projection membrane, and bBAF complex. Finally, for MF, protective proteins were enriched in neurexin family protein binding, ubiquitin-like protein transferase activity, and aminoacyltransferase activity pathways. According to KEGG enrichment analysis, these proteins were found to be enriched in B cell receptor signaling pathway, Natural killer cell-mediated cytotoxicity, Ubiquitin-mediated proteolysis, and cAMP signaling pathway.

## 4. Discussion

This study systematically investigates the causal relationships between 4907 circulating proteins and HF and AF based on a large-scale pQTL study and publicly available IEU open GWAS data. Utilizing SNPs as IVs and applying the criteria of *P*_IVW_ < .05 and *P*_FDR_ < .05, a total of 23 circulating proteins associated with the diseases were identified. Among them, THSD1 (*P*_IVW_ < .001, *P*_FDR_ = .002, OR = 0.920, 95% CI = 0.890–0.950), ALPI (*P*_IVW_ < .001, *P*_FDR_ = .003, OR = 0.868, 95% CI = 0.819–0.920), DEFB135 (*P*_IVW_ < .001, *P*_FDR_ = .017, OR = 0.782, 95% CI = 0.699–0.875), and L1CAM (*P*_IVW_ < .001, *P*_FDR_ = .017, OR = 0.862, 95% CI = 0.804–0.923) are protective proteins for HF; DEFB135 (*P*_IVW_ < .001, *P*_FDR_ = .004, OR = 0.809, 95% CI = 0.741–0.882), ARID1A (*P*_IVW_ < .001, *P*_FDR_ = .004, OR = 0.721, 95% CI = 0.629–0.826), KIR2DS4 (*P*_IVW_ < .001, *P*_FDR_ = .004, OR = 0.805, 95% CI = 0.735–0.883), PDE4D (*P*_IVW_ < .001, *P*_FDR_ = .004, OR = 0.807, 95% CI = 0.736–0.885), SYTL1 (*P*_IVW_ < .001, *P*_FDR_ = .005, OR = 0.734, 95% CI = 0.640–0.841), RLN2 (*P*_IVW_ < .001, *P*_FDR_ = .005, OR = 0.882, 95% CI = 0.834–0.933), CES1 (*P*_IVW_ < .001, *P*_FDR_ = .008, OR = 0.844, 95% CI = 0.780–0.912), VAV3 (*P*_IVW_ < .001, *P*_FDR_ = .015, OR = 0.812, 95% CI = 0.735–0.897), ANGPTL3 (*P*_IVW_ < .001, *P*_FDR_ = .018, OR = 0.915, 95% CI = 0.876–0.955), CD79B (*P*_IVW_ < .001, *P*_FDR_ = .020, OR = 0.777, 95% CI = 0.687–0.880), UBE2M (*P*_IVW_ < .001, *P*_FDR_ = .027, OR = 0.814, 95% CI = 0.733–0.904), IL17RC (*P*_IVW_ < .001, *P*_FDR_ = .027, OR = 0.713, 95% CI = 0.600–0.848), CASK (*P*_IVW_ < .001, *P*_FDR_ = .032, OR = 0.754, 95% CI = 0.651–0.873), HERC4 (*P*_IVW_ < .001, *P*_FDR_ = .036, OR = 0.823, 95% CI = 0.742–0.912), RND1 (*P*_IVW_ < .001, *P*_FDR_ = .036, OR = 0.844, 95% CI = 0.772–0.923), MDM4 (*P*_IVW_ < .001, *P*_FDR_ = .045, OR = 0.841, 95% CI: 0.766–0.923), CEACAM20 (*P*_IVW_ < .001, *P*_FDR_ = .046, OR = 0.742, 95% CI: 0.631–0.872), and HEPHL1 (*P*_IVW_ < .001, *P*_FDR_ = .047, OR = 0.847, 95% CI = 0.773–0.927) are protective proteins for AF; However, SLIT2 (*P*_IVW_ < .001, *P*_FDR_ = .004, OR = 1.218, 95% CI = 1.118–1.327) and FABP4 (*P*_IVW_ < .001, *P*_FDR_ = .027, OR = 1.299, 95% CI = 1.138–1.483) are risk proteins for AF.

It is noteworthy that DEFB135 emerges as a protective protein shared by HF and AF. Human defensins, classified into alpha-defensins, beta-defensins, and theta-defensins based on structural differences, play crucial roles in innate and adaptive immune responses.^[24]^ DEFB135, belonging to the beta-defensins family, exhibits significant antifungal, antibacterial, and antiviral activities, serving as a pivotal mediator in immune responses.^[25]^ The immunomodulatory functions mediated by beta-defensins include regulating inflammatory responses, neutralizing lipopolysaccharides, recruiting and activating immune cells, inducing cell proliferation, promoting wound healing, and maintaining skin barrier integrity.^[26]^ The intricate pathogenesis of HF implicates the cardiac immune microenvironment as a core factor in its progression.^[27]^ As a complex arrhythmia, AF’s onset, persistence, and complications involve multifactorial elements, with inflammation-mediated atrial structural remodeling being a critical precipitating factor. Previous studies have highlighted elevated levels of C-reactive protein in the serum of AF patients compared to those with normal sinus rhythm.^[28]^ While direct reports on DEFB135 remain scarce, studies have demonstrated other beta-defensins’ ability to downregulate the expression of inflammatory factors, promote vascular regeneration,^[29–31]^ induce vasodilation and lower blood pressure through immunological pathways,^[32]^ as well as induce fibroblast migration and proliferation, accelerating wound healing.^[25]^ Mediation MR analysis indicates that DEFB135 mitigates HF risk by reducing AF risk. Clinically, HF often represents the final outcome of cardiovascular diseases. Whether atherosclerosis or hypertension, both can predispose individuals to AF, subsequently impacting ventricular ejection capacity, leading to deteriorated cardiac function and eventual HF development.^[33]^ Thus, regulating immune responses in this process is crucial, with DEFB135 potentially serving as a novel target for intervening in HF and AF, although extensive experimentation is warranted to elucidate its specific mechanisms of action.

HF typically ensues as a symptom of ischemic heart disease (IHD) progression, making early intervention in IHD an effective means of preventing HF. Studies have indicated that high levels of ALPI may confer protective effects against IHD.^[34]^ Lipopolysaccharide (LPS) can induce inflammation in various tissues/cells, including cardiomyocytes, leading to diseases such as myocarditis and cardiac hypertrophy. Long noncoding RNA SOX2 overlapping transcript has been shown to protect cardiomyocytes from LPS-induced damage by modulating L1CAM.^[35]^ Furthermore, L1CAM has been identified as a protective factor against AF,^[36]^ corroborating the negative correlation between L1CAM and HF observed in our MR study. THSD1 is also considered a protective protein against HF, yet further experimental validation is required. Inflammation and fibrosis are key mechanisms in AF pathogenesis. In our MR analysis, SLIT2 and FABP4 were identified as risk proteins for AF, predicting disease progression. SLIT2 may promote inflammation by activating the nuclear factor-κB pathway,^[37]^ and its association with myocardial fibrosis progression has been reported.^[38]^ Despite lacking direct evidence of SLIT2’s promotion of AF, numerous studies have confirmed FABP4 as the best predictor of recurrent AF after radiofrequency ablation,^[39–41]^ consistent with our findings. PDE4D, a member of phosphodiesterases predominantly expressed in atrial myocytes, regulates cyclic adenosine phosphate levels and Ca^2+^ influx/release in atria, thereby preventing atrial arrhythmias.^[42]^ Studies have shown the correlation between RLN2 and AF, with RLN2 inhibiting atrial fibroblast migration, reducing inflammation, and exerting protective effects against AF.^[43]^ Additionally, RLN2 is a candidate drug for acute HF, enhancing myocardial contractility.^[44]^

Enrichment analysis reveals pathways closely associated with HF, including protease binding, magnesium ion binding, thiamin metabolism, and folate biosynthesis. For instance, ubiquitin-specific protease 4 and ubiquitin-specific protease 18 expressed in the heart inhibit cardiac hypertrophy and delay HF progression.^[45,46]^ The potential protective role of Mg^2+^ against HF and AF involves reducing oxidative stress, fibrosis, and electrical remodeling in the heart.^[47]^ Thiamine, also known as Vitamin B1, is essential for energy generation in myocardial contraction and holds significance in HF treatment.^[48]^ Folic acid’s cardioprotective effects prevent cardiac remodeling and dysfunction via endoplasmic reticulum stress pathways and delay cellular senescence.^[49]^ Pathways related to AF encompass cholesterol homeostasis, sterol homeostasis, and the cAMP signaling pathway. Obesity and metabolic syndrome are considered risk factors for AF, with meta-analyses showing increased AF incidence with elevated total cholesterol levels.^[50]^ The correlation between cyclic adenosine phosphate and AF, as discussed earlier, suggests PDE4D as a potential target for activating the cAMP signaling pathway.

Our study holds several strengths. Firstly, leveraging pQTL data from the largest protein GWAS to date ensures more reliable results than previous studies. Secondly, adhering to the MR core assumption, rigorous criteria were applied in IVs selection, greatly enhancing the study’s credibility. Thirdly, the identification of DEFB135 as a shared protective protein for HF and AF, along with the demonstration of AF mediation, adds novelty to our findings. Fourthly, the results of reverse MR analysis were not statistically significant, which further strengthens the persuasiveness of the forward MR analysis and affirms the potential of these circulating proteins as drug targets. Finally, enrichment analysis and drug target analysis, as additional analyses, augment the study’s comprehensiveness. However, our study also has limitations. Firstly, while using data solely from European populations for exposure and outcome minimizes bias due to population stratification, generalizability to patients in other regions may be limited. Secondly, enrichment analysis and drug target analysis are predictive analyses, necessitating validation of their accuracy. Lastly, the availability of larger sample databases in the future is anticipated to yield additional positive results.

## 5. Conclusion

In conclusion, our comprehensive MR analysis assesses the correlation between 4907 circulating proteins and HF and AF, demonstrating high credibility due to the rigorous study design and providing insights for future drug development.

## Acknowledgments

We thank all participants and investigator involved in the E. Ferkingstad et al genome-wide association analysis on proteome and the HERMES Consortium study for sharing data.

## Author contributions

**Conceptualization:** Weibo Zhong.

**Data curation:** Zheng Tang.

**Funding acquisition:** Dandan Guo.

**Investigation:** Yating Wang, Ruixue Zuo.

**Methodology:** Jixin Li.

**Project administration:** Dandan Guo.

**Resources:** Xiaohan Xiu.

**Software:** Zhenyu Yang.

**Supervision:** Dandan Guo.

**Validation:** Fengzhao Liu.

**Visualization:** Zhenyu Yang.

**Writing – original draft:** Zhenyu Yang, Xiaohan Xiu.

**Writing – review & editing:** Fengzhao Liu, Jixin Li, Zheng Tang, Weibo Zhong, Shoulei Xu, Shuo Ma, Yating Wang, Ruixue Zuo, Dandan Guo.

## Supplementary Material


