# Supplementary material for: Causal relationships between 4907 circulating proteins and heart failure and atrial fibrillation: A bidirectional Mendelian randomization study
Source: Medicine (Baltimore). 2025 Jul 4;104(27):e43198. doi: 10.1097/MD.0000000000043198 (PMC12237323; doi:10.1097/MD.0000000000043198)
Supplement: Supplementary file 2 [file medi-104-e43198-s002.docx]

**Table 25** Heterogeneity analysis and directional pleiotropy analysis.

| **Exposure** | **Outcome** | **Cochran’s *Q* analysis** | | **MR-Egger intercept test** | |
| --- | --- | --- | --- | --- | --- |
|  |  | **MR-Egger** | **IVW** | **Egger_intercept** | ***P* value** |
| ALPI | HF | *Q*=1.227 | *Q*=2.336 | 0.008 | 0.403 |
|  |  | *P*=0.541 | *P*=0.506 |  |  |
| DEFB135 | HF | *Q*=2.142 | *Q*=2.244 | -0.005 | 0.770 |
|  |  | *P*=0.544 | *P*=0.126 |  |  |
| L1CAM | HF | *Q*=12.447 | *Q*=15.156 | 0.008 | 0.195 |
|  |  | *P*=0.189 | *P*=0.126 |  |  |
| THSD1 | HF | *Q*=13.341 | *Q*=15.235 | 0.018 | 0.260 |
|  |  | *P*=0.205 | *P*=0.172 |  |  |
| ANGPTL3 | AF | *Q*=5.333 | *Q*=9.453 | -0.013 | 0.077 |
|  |  | *P*=0.722 | *P*=0.397 |  |  |
| ARID1A | AF | *Q*=1.800 | *Q*=1.803 | -0.001 | 0.964 |
|  |  | *P*=0.407 | *P*=0.614 |  |  |
| CASK | AF | *Q*=1.035 | *Q*=1.886 | 0.016 | 0.531 |
|  |  | *P*=0.309 | *P*=0.389 |  |  |
| CD79B | AF | *Q*=0.607 | *Q*=1.528 | 0.040 | 0.513 |
|  |  | *P*=0.435 | *P*=0.465 |  |  |
| CEACAM20 | AF | *Q*=1.254 | *Q*=2.189 | 0.017 | 0.547 |
|  |  | *P*=0.262 | *P*=0.335 |  |  |
| CES1 | AF | *Q*=0.701 | *Q*=3.873 | 0.031 | 0.149 |
|  |  | *P*=0.951 | *P*=0.568 |  |  |
| DEFB135 | AF | *Q*=0.846 | *Q*=1.298 | 0.008 | 0.549 |
|  |  | *P*=0.839 | *P*=0.862 |  |  |
| FABP4 | AF | *Q*=1.198 | *Q*=1.915 | 0.019 | 0.580 |
|  |  | *P*=0.274 | *P*=0.384 |  |  |
| HEPHL1 | AF | *Q*=0.159 | *Q*=1.981 | 0.034 | 0.270 |
|  |  | *P*=0.984 | *P*=0.739 |  |  |
| HERC4 | AF | *Q*=0.226 | *Q*=2.206 | 0.015 | 0.295 |
|  |  | *P*=0.893 | *P*=0.531 |  |  |
| IL17RC | AF | *Q*=0.461 | *Q*=1.219 | 0.013 | 0.544 |
|  |  | *P*=0.497 | *P*=0.544 |  |  |
| KIR2DS4 | AF | *Q*=2.549 | *Q*=3.121 | 0.007 | 0.504 |
|  |  | *P*=0.466 | *P*=0.538 |  |  |
| MDM4 | AF | *Q*=0.0857 | *Q*=2.289 | 0.134 | 0.377 |
|  |  | *P*=0.770 | *P*=0.318 |  |  |
| PDE4D | AF | *Q*=3.020 | *Q*=3.053 | 0.002 | 0.868 |
|  |  | *P*=0.389 | *P*=0.549 |  |  |
| RLN2 | AF | *Q*=6.346 | *Q*=6.386 | -0.002 | 0.852 |
|  |  | *P*=0.386 | *P*=0.495 |  |  |
| RND1 | AF | *Q*=4.150 | *Q*=4.298 | 0.012 | 0.765 |
|  |  | *P*=0.246 | *P*=0.367 |  |  |
| SLIT2 | AF | *Q*=8.223 | *Q*=8.499 | -0.006 | 0.670 |
|  |  | *P*=0.222 | *P*=0.291 |  |  |
| SYTL1 | AF | *Q*=0.017 | *Q*=0.583 | 0.013 | 0.589 |
|  |  | *P*=0.896 | *P*=0.747 |  |  |
| UBE2M | AF | *Q*=0.716 | *Q*=1.280 | -0.015 | 0.590 |
|  |  | *P*=0.397 | *P*=0.527 |  |  |
| VAV3 | AF | *Q*=2.219 | *Q*=2.666 | 0.007 | 0.591 |
|  |  | *P*=0.330 | *P*=0.446 |  |  |
